# Father trait anger: Associations with father–infant bonding and subsequent parenting stress

**DOI:** 10.3389/fpsyg.2023.1114084

**Published:** 2023-03-10

**Authors:** Lauren M. Francis, George J. Youssef, Christopher J. Greenwood, Peter G. Enticott, Ashlee Curtis, Liam G. Graeme, Kayla A. Mansour, Craig A. Olsson, Helen Skouteris, Jeannette Milgrom, Joanne Williams, Tess Knight, Jacqui A. Macdonald

**Affiliations:** ^1^Centre for Social and Early Emotional Development, School of Psychology, Faculty of Health, Deakin University, Geelong, VIC, Australia; ^2^Centre for Adolescent Health, Murdoch Children’s Research Institute, Royal Children’s Hospital, Melbourne, VIC, Australia; ^3^Centre for Drug Use, Addictive and Anti-social Behaviour Research, School of Psychology, Deakin University, Geelong, VIC, Australia; ^4^Department of Paediatrics, Faculty of Medicine, Dentistry and Health Sciences, University of Melbourne, Parkville, VIC, Australia; ^5^Health and Social Care Unit, School of Public Health and Preventive Medicine, Monash University, Melbourne, VIC, Australia; ^6^Warwick Business School, Warwick University, Coventry, United Kingdom; ^7^Parent-Infant Research Institute, Austin Health, Heidelberg, VIC, Australia; ^8^Melbourne School of Psychological Sciences, The University of Melbourne, Melbourne, VIC, Australia; ^9^Department of Health Sciences and Biostatistics, Swinburne University of Technology, Hawthorn, VIC, Australia; ^10^School of Health and Social Development, Deakin University Faculty of Health, Burwood, VIC, Australia; ^11^Cairnmillar Institute, Hawthorn East, VIC, Australia

**Keywords:** fathers, infants, children, anger, bonding, parenting stress, longitudinal

## Abstract

**Introduction:**

Parent anger presents a risk to family safety and child development. Father trait anger may also compromise the early relational context of fathers and offspring, yet evidence is lacking. The aim of this study is to examine effects of father trait anger on parenting stress in the toddler years, and the mediational role of father–infant bonding.

**Method:**

Data were from 177 Australian fathers of 205 children. Trait anger (total, angry temperament, and angry reaction), father–infant bonding subscales (patience and tolerance, affection and pride, and pleasure in interaction), and subsequent parenting stress (parental distress, difficult child, and parent–child dysfunctional interaction) were assessed. At each of the subscale levels, mediational path models examined whether father–infant bonding explained the relationship between trait anger and parenting stress. Models were presented where there was at least a small association between the mediator and both the predictor and outcome.

**Results:**

Patience and tolerance was the only domain of father–infant bonding correlated with both trait anger and all parenting stress outcomes. Patience and tolerance partially mediated the effect of total trait anger on parental distress and fully mediated effects on difficult child and parent–child dysfunctional interaction. Patience and tolerance fully mediated relationships between angry temperament and all domains of parenting stress. Angry reactions only had a direct effect on parental distress.

**Discussion:**

Father trait anger both directly and indirectly (through patience and tolerance in the father–infant bond) impacts their experiences of parenting stress in the toddler years. Early interventions to manage father trait anger and improve father–infant bonding may benefit fathers and children.

## 1. Introduction

Trait anger is the propensity to perceive situations or stimuli as annoying or frustrating and to respond with emotion states that extend from irritation and annoyance, to fury and rage (Spielberger et al., 1995). Approximately 8% of the population experience elevated trait anger at levels that interfere with general functioning (Okuda et al., 2015). Prevalence is higher in men than women and highest across young adulthood (Okuda et al., 2015), which coincides with the peak age for the transition to fatherhood (Australian Bureau of Statistics, 2016; U.S. Census Bureau, 2019). In the family environment, father anger presents a risk to family safety (Mammen et al., 2000; Stith et al., 2009) and has been shown to have long-term detrimental effects on children’s social and emotional development, including children’s angry and aggressive behaviors (Conger et al., 2003; Dayton and Malone, 2017). Trait anger is linked to an increased likelihood of automatically appraising others as having hostile intentions (Li and Xia, 2020), and a reduced likelihood of reappraising those judgments (Prikhidko and Swank, 2019). In these ways, high levels of trait anger in fathers may cast a dispositional filter that increases negative appraisals of child behaviors and triggers angry emotions and a desire to act on those emotions verbally or physically. Despite the potential for harm within families, research on father trait anger is scarce and lacks investigations into longitudinal outcomes.

One of the earliest indicators of parents’ adjustment to the parenting role that may be compromised by trait anger is the capacity to form a bond with their infants. The Paternal Postnatal Attachment Scale (PPAS) is one of the most common measures of the father–infant bond and conceptualizes fathers’ feelings of bonding across three domains; affection and pride in the infant, pleasure in their interactions, and patience and tolerance in the context of the demands of the role (Condon et al., 2008). Fathers’ bonds with infants typically develop over the course of the first postpartum year and indicate a capacity to be a secure and reliable attachment figure (Condon et al., 2013; Aytuglu and Brown, 2022). Although no research, to our knowledge, has investigated associations between a propensity to feel anger (i.e., trait anger) and father–infant bonding, studies have linked state anger (i.e., feeling angry in the moment; Spielberger, 1999) and related constructs to deficits in parent–infant relationships. For example, father state anger, in combination with depressive symptoms, has been shown to predict poorer postpartum father–infant bonds (Macdonald et al., 2020). Further, harsh parenting, characterized by angry and hostile behaviors toward one’s own child, is associated with reduced closeness in parent–child relationships generally (Chung et al., 2022), and attachment insecurity in father–child relationships specifically (Wang et al., 2021). Men’s violence toward intimate partners during pregnancy is also linked to weaker emotional bonds with their infants (Nishigori et al., 2020).

In the subsequent toddler years, the enduring nature of trait anger likely has a pervasive impact on the father–toddler relationship. The Parenting Stress Index-3 Short Form (PSI-SF) measures the experience of stress within the parenting role and includes characterization of the parent–child relationship (Abidin, 1995). It is among the most widely used indicators of concerns in the parent–child relationship during the toddler years (Barroso et al., 2018). The PSI-SF conceptualizes parenting stress across three domains: feelings of distress within the parenting role, appraisals of children as being difficult, and patterns of negative interactions with children (Abidin, 1995; Lee et al., 2016). Prior literature has found state anger/hostility to be associated with parenting stress during the infancy years specifically in mothers (Georg et al., 2021) and in the subsequent early to mid-childhood years in samples of mothers and fathers (Lam, 1999; Rodriguez and Richardson, 2007; Chung et al., 2022).

Taken together, evidence is emerging of links between anger and both poorer father–infant bonding in infancy and increased parenting stress in the toddler years. Further, poor father–infant bonding has been prospectively linked to subsequent parenting stress (de Cock et al., 2017), suggesting a possible mediational pathway that is yet to be explored. It is plausible that father trait anger may fuel higher levels of parenting stress in the toddler years and that deficits in establishing a strong emotional father–infant bond may explain some or all of that relationship. That is, trait anger may undermine formation of a stable bond which may lead to greater stress within the father–toddler relationship.

The current literature is limited by a focus on state anger, predominantly investigated in cross-sectional designs. It therefore does not reveal the degree to which a father’s propensity for anger underlies the situational angry affect. This is important to investigate as trait anger in men may account for stable and enduring risk within families. One reason that trait anger may have been neglected in prior family studies may be because researchers tend to focus on factors they deem to be malleable and therefore optimal targets for intervention. Trait anger’s stability is attributed to both genetic and early environmental origins (Rebollo and Boomsma, 2006). However, traits are maintained by self-reinforcing cognitions and actions (Izard, 1993; Zimprich and Mascherek, 2012), and evidence suggests that these patterns offer potential points of modification. Evidence exists of trait anger levels being reduced by interrupting self-reinforcing cycles with Cognitive Behavioral Therapy and mindfulness interventions (Rodriguez Vega et al., 2014; Fernandez et al., 2018). Although interventions often require intensive treatment across a long program of sessions (e.g., 12–14 weeks, see Korman et al., 2008; Hutchinson et al., 2017), significant and clinically meaningful reductions in trait anger have been found in shorter intervention programs, even in samples with elevated levels of trait anger (e.g., 2–4 weeks, see Howie and Malouff, 2014; Vassilopoulos et al., 2015). In the context where trait anger in men may be negatively impacting relationships with offspring and parenting stress, intervention at the earliest point (i.e., pre-conception or during pregnancy) may prevent or disrupt feedback loops of negative interactions that reinforce the trait affective responses and frequency of behavioral expression of anger.

An additional consideration in targeting trait anger for possible intervention is that it comprises two distinct facets. These are trait *angry temperament*, which is the tendency to experience anger without necessarily being provoked by a stimulus or situation, and trait *angry reaction*, which is the frequency with which angry feelings are experienced in response to frustrating situations or stressors (Spielberger et al., 1995; Lievaart et al., 2016). A tendency to react with anger and often feel angry regardless of provocation are both likely to impact the father–infant bond and parenting stress in the toddler years. However, though these facets are strongly correlated (Lievaart et al., 2016), they remain distinct. This is evident in differential associations across mental health presentations. For example, both facets are similarly associated with depression, yet only angry reaction is associated with attention-deficit/hyperactivity disorder (Lubke et al., 2015). Such discrete associations for the facets may also emerge in the parenting context, however, this is yet to be examined. Knowledge of facet-level associations of trait anger is relevant to anger treatment approaches where therapeutic strategies for a father who frequently feels angry regardless of provocation may differ to those adopted with fathers whose anger is primarily in response to provocation. Empirical evidence supports targeted treatments with efficaciousness varying by facet-treatment combinations in non-parenting contexts (see Rodriguez Vega et al., 2014; Kubiak et al., 2015). Thus, investigation of the impact of both father angry temperament and reactions on father–infant bonding and parenting stress is warranted.

If early detection of trait anger is missed, or men are not receptive to intervention before becoming fathers, the parent–infant bond is a modifiable option for postpartum intervention to potentially prevent future parenting stress in the toddler years (Mascheroni and Ionio, 2019). Identification of multiple potential targets and time points may help to best tailor interventions to provide new fathers with the opportunity to establish close relationships with children grounded in patterns of positive interactions. This is particularly important given that the extent to which men, particularly those with high levels of trait anger, will engage with interventions preconception is unknown (Toivonen et al., 2017).

Despite the importance of the father–child relationship (Sarkadi et al., 2008; Baldwin et al., 2018; Cabrera et al., 2018), existing research predominantly focuses on the mother–child relationship (Cabrera, 2020). In the context of gender-socialized caregiving roles and limited paternal leave, father–infant relationships face unique challenges, including a delay in opportunities for father–infant bonding at birth and postpartum compared to mothers (Scism and Cobb, 2017). The nature of father–toddler interactions also differs from mother–toddler interactions. Fathers more often than mothers engage in challenging rough and tumble play with their children, which, when engaged in sensitively, is argued to be a contributing factor in the child’s developing self-regulation, social development, and emotion regulation (StGeorge and Freeman, 2017; Amodia-Bidakowska et al., 2020). Due to these differences between father- and mother–child relationships and their experience of anger, it is important to investigate fathers’ experiences specifically.

The aim of the current study was to draw on rare longitudinal data from men in the early years of fatherhood to examine effects of father trait anger on father–infant bonding and subsequent parenting stress in the toddler years. It was hypothesized that levels of trait anger would be positively associated with parenting stress one to two years later during toddlerhood and that father–infant bonding would mediate that association. Variation in strengths of pathways, indicated by factors of trait anger, bonding, and parenting stress, were explored.

## 2. Methods

### 2.1. Participants

Participants were from the Men and Parenting Pathways study (MAPP, *N* = 608), a cohort study investigating the mental health and wellbeing of men with and without children during the peak age for first-time fatherhood in Australia (Australian Bureau of Statistics, 2016; Macdonald et al., 2021). Ethics approval was granted by Deakin University (HEAG-H 192_2014). MAPP commenced in 2015 with annual data collection. At the time of analysis, four waves of data collection were complete. The MAPP sample was recruited through social media, partnership organizations, and word of mouth. MAPP eligibility included: aged 28–32 years at commencement, male, English-speaking, and an Australian resident. Eligible participants were asked to provide informed consent for participation for the first survey and for the following waves of data collection.

In line with the recruitment protocols of the Australian Longitudinal study on Women’s health (Loxton et al., 2015), recruitment was successful in reaching representativeness across a number of key demographics (Macdonald et al., 2021). The MAPP sample did not differ significantly from the spread of socio-economic advantage and disadvantage across areas (postcodes) of Australia, as designated by the Australian Bureau of Statistics (2018). A similar proportion of men in MAPP had completed post high-school education compared to males of the same age in the Australian population (Australian Bureau of Statistics, 2019). The MAPP sample included a representative number of participants of Aboriginal or Torres Strait Islander background (2%; Australian Bureau of Statistics, 2017a). There were slightly more men in MAPP who identified as non-heterosexual than Australian rates in 2014 (Australian Bureau of Statistics, 2015), one year before initial data collection for the MAPP study; however; the number of men aged 25 to 39 who identify as non-heterosexual has increased in recent years (Australian Bureau of Statistics, 2021). Compared to 2016 Australian census data (Australian Bureau of Statistics, 2017b), there were fewer men in the MAPP sample born outside Australia; however, this may be partly because inclusion criteria for the MAPP study required participants to be Australian residents, whereas the census more broadly included all people in Australia on the census date including residents, non-residents, and visitors. For additional detail, see the MAPP Study cohort profile (Macdonald et al., 2021). Additionally, no differences were identified between the full MAPP sample and participants who responded at Waves 2 or 3 on key demographic characteristics assessed at baseline (Macdonald et al., 2021).

Fathers eligible for inclusion were those who had an infant at any of the first three waves of data collection (defined as under 12-months-of-age at Wave 1 or under 18-months at Waves 2 or 3) and who had data on at least one of the predictor, mediator, or outcome variable; *n* = 177 fathers of 205 infants. For a discussion of power and how sample size was determined for the full MAPP cohort see Macdonald et al. (2021). For convention, power analysis for the analytic sample is reported here. Power was calculated based on detecting moderate effect sizes, which were equivalent or more conservative than those found in prior research (de Cock et al., 2017; Chung et al., 2022): predictor-to-mediator, predictor-to-outcome, and mediator-to-outcome path effect sizes were specified as β = −0.25, β = 0.20, β = −0.35, respectively. The size of the standardized indirect effect was thus estimated to be small (β = 0.087). Using Monte Carlo simulation in Mplus (Muthén and Muthén, 2015), a sample size of 177 participants was found to provide 88% power to detect the specified indirect effect, at alpha = 0.05. Therefore, the current study was sufficiently powered to detect indirect effects of interest.

### 2.2. Measures

#### 2.2.1. Trait anger

The State–Trait Anger Expression Inventory-2 (STAXI-2; Spielberger, 1999) was used to measure *total trait anger* and its facets, trait *angry temperament* and trait *angry reaction,* in MAPP only in Wave 1. The 10-item total trait anger scale comprises four items from the *angry temperament* subscale (e.g., “I am a hotheaded person”), four items from the *angry reaction* subscale (e.g., “It makes me furious when I am criticized in front of others”), and an additional two items which are unique to the total scale (e.g., “When I get frustrated, I feel like hitting someone”). Response options range from 1 (*Almost never*), to 4 (*Almost always*). Factor structure and validity of the STAXI-2 trait anger scales have been demonstrated in males (Spielberger, 1999; García-León et al., 2002; Lievaart et al., 2016). The trait anger scale had good internal consistency in the MAPP sample (α = 0.88). Higher scores indicate higher levels of trait anger.

#### 2.2.2. Father–infant bond

The PPAS (Condon et al., 2008; Condon, 2015) was administered to participants reporting to be a father of an infant up to 12-months-of-age at Wave 1, and up to 18-months-of-age at Waves 2 and 3, with the extended age range to accommodate participants who were delayed in completing their annual survey. The subscales are: the 8-item *patience and tolerance* subscale (e.g., “When I am caring for the baby I get feelings that the child is deliberately being difficult or trying to upset me”), the 7-item *pleasure in interaction* subscale (e.g., “I try to involve myself as much as possible in child care and looking after the baby”), and the 4-item *affection and pride* subscale (e.g., “When I am with the baby and other people are present, I feel proud of the baby”). Response options include item-specific 3, 4, and 5-point Likert scales, and true/untrue contrasts, which are weighted to an equivalent 1- to 5-point scale for analysis. The PPAS has previously been used with fathers of children aged up to 24-months-of-age (de Cock et al., 2017). The PPAS has acceptable validity and reliability (Condon et al., 2008) and good internal consistency in the MAPP sample (α = 0.83 to 0.87). Higher scores indicate higher levels of bonding.

#### 2.2.3. Parenting stress

The PSI-SF was used to measure three domains of stress in the father–toddler relationship across three 12-item subscales one year after assessment of the father–infant bond, i.e., in Waves 2, 3, and/or 4 (Abidin, 1995). In the MAPP study, the PSI-SF was assessed only in fathers of children older than 12 months with consideration of participant burden on those with children younger than 12 months who were already answering the PPAS father–infant bonding measure. The first PSI-SF subscale, *parental distress,* reflects general personal distress (e.g., “I feel trapped by my responsibilities as a parent”). Two further subscales reflect childrearing stress indicated by reports of having a *difficult child* (e.g., “My child seems to cry or fuss more often than most children”) and *parent–child dysfunctional interaction* (e.g., “My child rarely does things for me that make me feel good”). Thirty-four of the items are anchored from 1 (*Strongly disagree*) to 5 (*Strongly agree*), the remaining two items have item-specific response anchors on a 5-point Likert scale. The PSI-SF is validated for use with parents of children aged from 1 month up to 12 years (Abidin, 1995) and has excellent internal consistency in the MAPP sample (α = 0.93 to 0.94). Higher scores indicate higher levels of parenting stress. In the current study, fathers’ PSI-SF data were taken from the next survey following assessment of father–infant bonding.

#### 2.2.4. Potential confounding variables

Covariates were included where they could theoretically cause both the predictor and outcome (Rohrer, 2018). Participant covariates measured at Wave 1 included household income (0 = *greater than or equal to $1,150 AUD*, 1 = *less than $1,150 AUD*), education (0 = *less than or equal to high school completion*, 1 = *greater than a high school education*), country of birth (0 = *Australia*, 1 = *not Australia*), and parental separation, divorce, or deceased before age 16 (0 = *no*, 1 = *yes*). Covariates measured at the mediator time points included child age (months), child sex (0 = *male*, 1 = *female*), and parent of only one child (0 = *yes*, 1 = *no*, father of more than one child). We also controlled for the wave of mediator assessment (0 = Wave 2 or 3, 1 = Wave 1) on the path between the predictor and the mediator. This was to account for the 33.2% of father–child dyads for whom an eligible child was already born at Wave 1, and therefore the predictor (trait anger) was measured concurrently with the mediator (father–infant bonding).

### 2.3. Analytic strategy

Data cleaning and descriptive statistics were conducted in Stata 15.1 (StataCorp, 2017). Summary statistics (i.e., means and standard deviations) and inferential analyses were conducted in Mplus Version 7.2 (Muthén and Muthén, 2015). Path models are presented for which there was indication of at least a small association (*r* > 0.10; Cohen, 1992) between the predictor and mediator and between the mediator and outcome. Path models were conducted for each combination of predictor (trait anger) and mediator (father–infant bond), with outcomes (parenting stress) explored simultaneously within models. Mediation was examined by simultaneously regressing: (1) the mediator (father–infant bond) on the predictor (trait anger) and covariates (household income, education, country of birth, parental separation, divorce, or deceased, and wave of mediator assessment); and (2) the outcomes (parenting stress) on the predictor (trait anger), mediator (father–infant bond), and covariates (household income, education, country of birth, parental separation, divorce, or deceased, child age, child sex, and parent of one child). Child-related covariates were included only at time points after the birth of the child. Mediation paths were estimated using a product of coefficients approach with the Mplus command “Model Indirect” (Rijnhart et al., 2019). In all analyses, standardized coefficients were specified with the “STDYX” Mplus command, full information maximum likelihood estimation with robust standard errors was used to address missing data, and cluster robust standard error estimators were used to account for the clustered nature of the data (i.e., fathers with multiple children).

## 3. Results

### 3.1. Descriptive statistics

The mean age of men in this sample was 30.05 (*SD* = 1.36) at time of predictor assessment. The mean age of the infant at the time of the mediator assessment (father–infant bonding) was 6.41 months (*SD* = 3.67) and at the time of outcome assessment (parenting stress) during toddlerhood was 19.10 months (*SD* = 3.81). Table 1 presents additional descriptive statistics of participants in the analytic sample.

**Table 1:** Descriptive characteristics of the analytic sample.

Variable	*n*	*%*
Father-related variables (*n* = 177)
In a relationship	175	98.9
Education > year 12	150	84.7
Household income ≥ $1,150 AUD weekly	158	89.3
Child-related variables (*n* = 205)
Biological parent of child	204	99.5
Child male	109	53.2
Parent of only one child	95	46.3

Participant-related variables measured at predictor time point. Child-related variables measured at mediator time point. No missing data on any descriptive variables.

Table 2 presents summary statistics and correlations between the key study variables. Total trait anger and its facets had small to medium associations with all domains of parenting stress in toddlerhood (*r* = 0.10 to 0.27) and with the patience and tolerance component of father–infant bonding (*r* = −0.13 to −0.21), but no associations with the pleasure in interaction and affection and pride components of father–infant bonding (*r* = 0.00 to −0.08). All components of father–infant bonding had medium to large associations with all domains of parenting stress (*r* = −0.33 to −0.57). Missing data on the predictor, mediator, and outcome variables ranged from 5.1 to 21.5%.

**Table 2:** Means, standard deviations, and correlations of key study variables.

Variables	*M*	SD	1	2	3	4	5	6	7	8
Trait anger
1. Total trait anger	17.78	6.26	–							
2. Angry temperament	6.26	2.91	0.89***	–						
3. Angry reaction	8.55	3.19	0.86***	0.57***	–					
Father–infant bonding
4. Patience and tolerance	30.89	5.01	−0.21**	−0.19**	−0.13	–				
5. Pleasure in interaction	24.98	4.77	−0.06	−0.08	0.01	0.59***	–			
6. Affection and pride	18.25	2.45	−0.08	−0.06	0.00	0.54***	0.64***	–		
Parenting stress
7. Parental distress	31.2	11.17	0.27***	0.21**	0.23**	−0.57***	−0.38***	−0.35***	–	
8. Difficult child	26.69	11.05	0.19*	0.21**	0.10	−0.51***	−0.41***	−0.33***	0.55***	–
9. Parent–child dysfunctional interaction	18.76	6.76	0.18*	0.16*	0.11	−0.49***	−0.45***	−0.55***	0.59***	0.69***

Cluster robust standard error estimators were used to account for the clustered nature of the data, i.e., fathers with multiple children. *p < 0.05, **p < 0.01, ***p < 0.001.

### 3.2. Mediation path models

Given the correlational evidence that patience and tolerance was the component of father–infant bonding associated with trait anger, mediation analyses are presented in this manuscript with this bonding component as the mediator.

**Figure 1:**
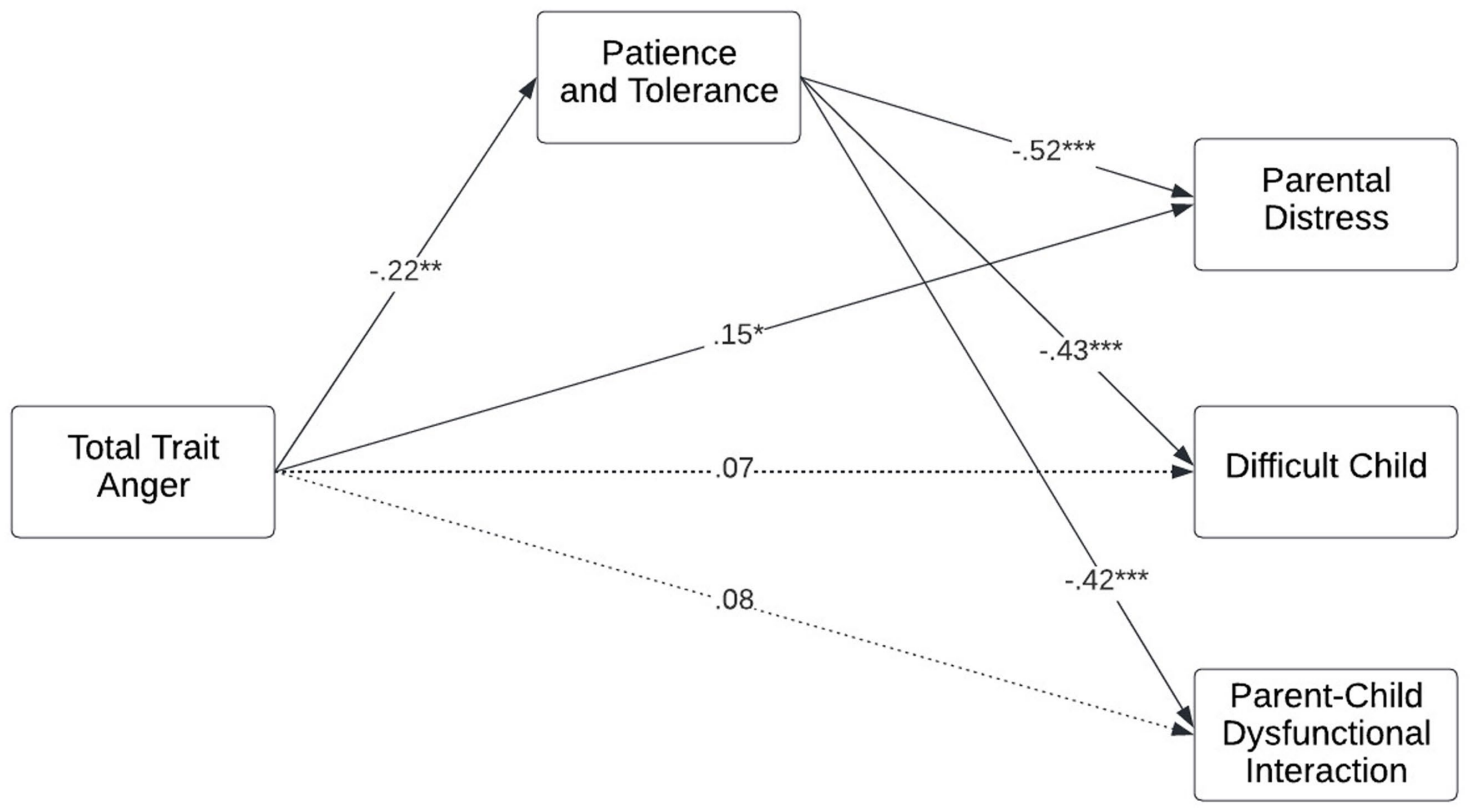
Father trait anger on parenting stress in the toddler years, through patience and tolerance in the father–infant bond. All effects are standardized. Model adjusted for covariates. *n* = 177 fathers of 205 children. Cluster robust standard error estimators were used to account for the clustered nature of the data, i.e., fathers with multiple children. Dotted line indicates non-significant paths. *p < 0.05, **p < 0.01, ***p < 0.001.

Figure 1 presents the mediational path model of total trait anger predicting domains of parenting stress, mediated by patience and tolerance. Patience and tolerance in father–infant bonding was found to partially mediate (i.e., significant indirect and direct effects) the effect of total trait anger on parental distress (indirect β = 0.12, *p* = 0.006; direct β = 0.15, *p* = 0.035) and fully mediate (i.e., significant indirect effect and non-significant direct effect) its effects on both difficult child (indirect β = 0.10, *p* = 0.017; direct β = 0.07, *p* = 0.299) and parent–child dysfunctional interaction (indirect β = 0.09, *p* = 0.024; direct β = 0.08, *p* = 0.371).

**Figure 2:**
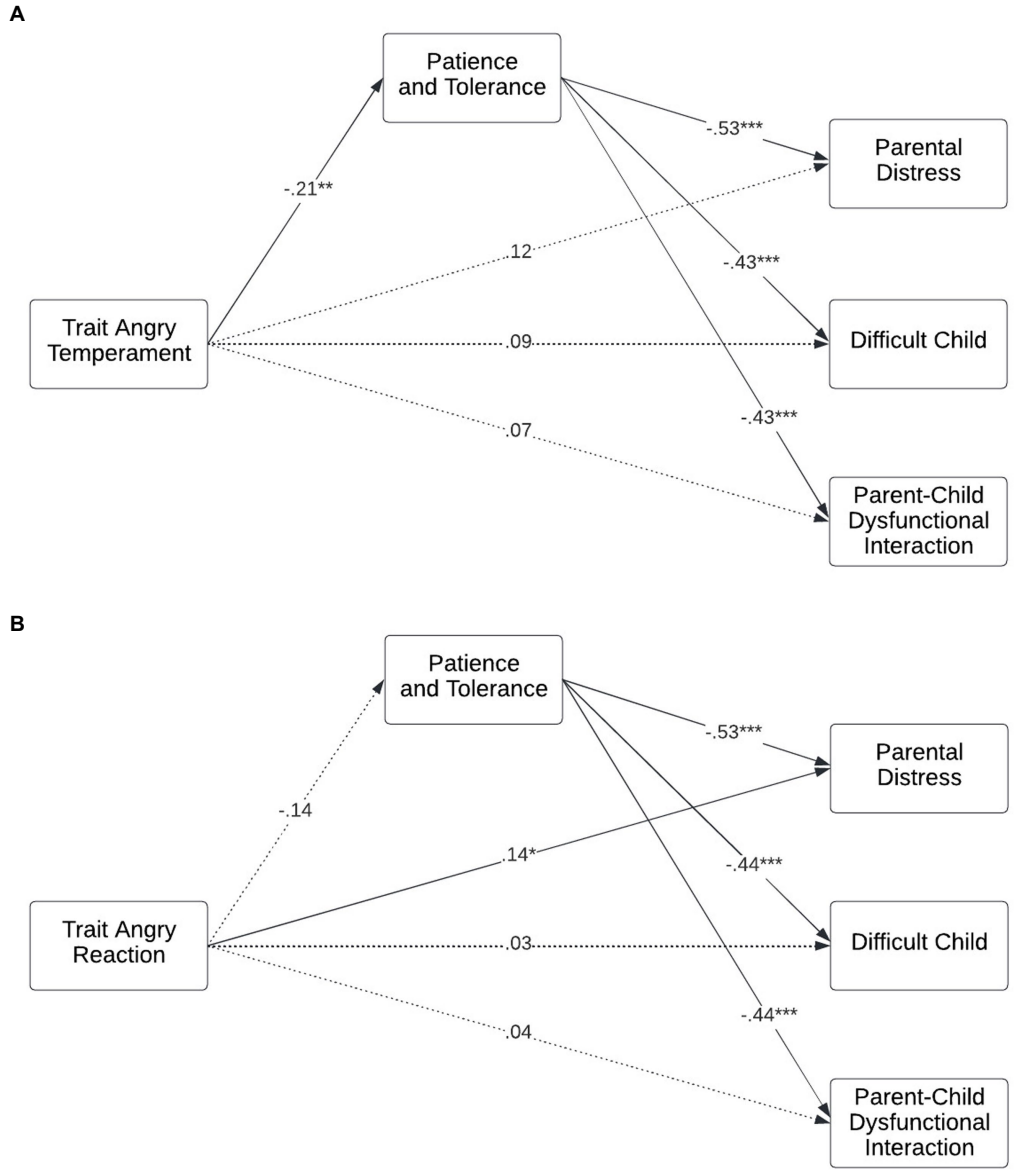
Father trait angry temperament **(A)** and trait angry reaction **(B)** on parenting stress in the toddler years, through patience and tolerance in the father–infant bond. All effects are standardized. Models are adjusted for covariates. *n* = 177 fathers of 205 children. Cluster robust standard error estimators were used to account for the clustered nature of the data, i.e., fathers with multiple children. Dotted line indicates non-significant paths. *p < 0.05, **p < 0.01, ***p < 0.001.

Further exploration of these relationships by facet of trait anger was then explored, see Figure 2. Patience and tolerance fully mediated the relationships between trait angry temperament and all three domains of parenting stress (Figure 2A); parental distress (indirect β = 0.11, *p* = 0.006; direct β = 0.12, *p* = 0.070), difficult child (indirect β = 0.09, *p* = 0.016; direct β = 0.09, *p* = 0.205), and parent–child dysfunctional interaction (indirect β = 0.09, *p* = 0.026; direct β = 0.07, *p* = 0.362). Trait angry reaction (Figure 2B) had only a direct effect on parental distress (indirect β = 0.07, *p* = 0.108; direct β = 0.14, *p* = 0.035). Trait angry reaction had no effect on perceptions of the child as difficult (indirect β = 0.06, *p* = 0.135; direct β = 0.03, *p* = 0.710) or dysfunctional interaction with that child (indirect β = 0.06, *p* = 0.149; direct β = 0.04, *p* = 0.648).

## 4. Discussion

The current study addressed a gap in knowledge about the pathways through which trait anger in men impacts their relationships with their infants and their subsequent parenting stress. We found effects of trait anger (total) on all domains of parenting stress in the toddler years; distress in their parenting role, perception of their toddler as difficult, and negatively charged interactions with their toddler. These effects were explained, at least in part, by levels of paternal patience and tolerance in the father–infant bond one year earlier. When investigated at the facet level of trait anger, more nuanced information about the pathways emerged. Specifically, patience and tolerance in the father–infant bond fully mediated associations between angry temperament and all three domains of parenting stress. By contrast, angry reactions had only a direct effect on one domain of parenting stress: parental distress. Although different pathways were revealed, our findings suggest that attention to both trait-based angry temperament and angry reactions may be warranted to mitigate fathers’ parenting stress in the toddler years.

Effects of having an angry temperament on all three domains of fathers’ parenting stress were fully mediated by patience and tolerance felt toward the child as an infant. These findings suggest that dispositional angry temperament in men may undermine formation of a father–infant bond, manifesting as reduced feelings of patience and tolerance. This appears to subsequently influence levels of stress in the father–toddler dyad, evident in fathers’ own distress, their critical perceptions of their toddlers’ behaviors, and of negative interactions with the child. Conversely, no mediation by any component of father–infant bonding was detected in the association with angry reactions. The presence of a mediation effect only for angry temperament may be because there is a clear theoretical alignment between angry temperament and the patience and tolerance component of the father–infant bond. This bonding component represents a father’s feelings of irritation, resentment, and low patience, embedded in the affective tie felt with that infant (Condon et al., 2008). Thus, patience and tolerance may be most aligned to the angry temperament component of trait anger which is felt across situations compared to the tendency to respond with anger to a stressor or trigger (i.e., angry reactions). Our findings provide some insights into antecedents of the previously reported prospective association between paternal bonding in infancy and parenting stress in toddlerhood (de Cock et al., 2017) and further deconstruct the relative importance of bonding components in these longitudinal pathways.

In analyses examining fathers’ tendencies for angry reactions, we found only a direct association with later parental distress during the toddler years. Consistent with this direct effect are prior studies that show that dispositional angry reactions are associated with a range of indicators of negative affect (Erwin et al., 2003). A possible explanation of this direct effect is that both angry reactions and parental distress reflect a reduced capacity for emotional-regulation (Wilkowski and Robinson, 2008; Hu et al., 2019). Encouragingly, we found that fathers’ trait angry reactions were not related to any component of the father–infant bond or to the two parenting stress subscales that specifically reflect childrearing stress in the toddler years (i.e., difficult child and parent–child dysfunctional interaction). Previous research on mothers identified that irritability, anger, and hostility were associated with increased parenting stress (Georg et al., 2021). These findings suggest more narrow effects in fathers identifying that a tendency for angry reactions specifically predicts parental distress where fathers report feeling trapped, overwhelmed, and lacking enjoyment in life.

Parental experiences of stress pervasively impact on the parenting context that the child develops within (Neece et al., 2012; Ward and Lee, 2020). Identification of trait anger as an antecedent of parenting stress adds important evidence to determine families that may be at risk and ascertain potential points of intervention. Trait anger is modifiable through intervention (Rodriguez Vega et al., 2014; Fernandez et al., 2018). Our findings suggest that if interventions with men, preconception or during pregnancy can reduce levels of angry temperament specifically, the flow-on effects may include increased feelings of patience and tolerance with infants and potentially lower parenting stress during the toddler years. However, due to the direct effect of angry reactions onto parental distress, pre-conception interventions may be most successful in preventing parenting stress in toddlerhood when they also target angry reactions, by both challenging existing thinking patterns that lead to a ‘build up’ of anger and by practicing strategies to diffuse anger in the moment (such as Naeem et al., 2008; Vassilopoulos et al., 2015; Hutchinson et al., 2017). However, research testing such an intervention is warranted, as the effectiveness of interventions on reducing each facet of trait anger is often not reported and when it is, the findings on the effectiveness in reducing both facets are equivocal (for example: Rodriguez Vega et al., 2014; Kubiak et al., 2015). Furthermore, most evidence on effectiveness of intervening on trait anger comes from offending or antisocial populations and whether these interventions are effective across a spectrum from moderate to high levels of trait anger or in fathers specifically is not clear (Fernandez et al., 2018).

Few interventions exist that target soon-to-be fathers (Goldstein et al., 2020), and the degree to which men would be motivated to engage in preconception behavior change is unknown (Toivonen et al., 2017). It may therefore be that the postpartum period is the first opportunity for intervention when men are motivated to engage in behavior change for the benefit of their new families. Evidence demonstrates that fathers are more engaged in anger-based interventions, such as treatments to reduce intimate partner violence, than men who are not fathers (Poole and Murphy, 2019). Therefore, if future research finds that men are not engaged in programs to reduce their anger pre-conception, then early fatherhood may be the best point to intervene to break the nexus between trait anger and parenting stress in the toddler years. Additionally, interventions to protect against parenting stress may be most successful if they target both trait anger and the father–infant bond (i.e., how to manage anger generally and specifically with their infant), as there is evidence that appropriate postpartum intervention can promote parent–infant bonding (Mascheroni and Ionio, 2019).

An additional finding of this study was that trait anger in men did not pose a risk to all components of father–infant bonding. At a bivariate level, trait anger was linked to lower patience and tolerance but had negligible associations with both affection and pride and pleasure in interaction in the father–infant bond. This finding is a positive message: a father’s high level of trait anger does not appear to be linked to his capacity to develop the components of a bond with his infant that involve warm emotions and pleasurable experiences.

### 4.1. Strengths and limitations

Due to the longitudinal nature of the study, missing data had the potential to introduce bias into estimates. Full information maximum likelihood estimation was used to address missing values, which has been shown to result in unbiased estimates in random-effects models (Schminkey et al., 2016). While there was some loss to follow-up, cohort studies with male participants tend to have lower retention rates than seen in the current study (Teague et al., 2018). Moreover, in the full MAPP sample, there were no key demographic differences between participants at Wave 1 and those who responded at Waves 2 or 3 (Macdonald et al., 2021). This study may also be limited by the use of self-report measures, which can be impacted by social desirability bias (van de Mortel, 2008). However, the STAXI-2 is the current standard measure of trait anger, and our sample does not appear to be affected substantially by social desirability bias evident in levels of trait anger being within two scale points of the normed data for males aged 30 years and older (Spielberger, 1999). Self-report methods can also be impacted by shared method variance. For instance, there is some conceptual overlap between father–infant bonding measured by the PPAS and parenting stress in the father–toddler relationship measured by the PSI-SF, as they both assess aspects of the parent–child relationship. However, the temporal separation of bonding (during infancy) and parenting stress (1 year later in the toddler years) indicators enables examination of the effect of trait anger on the father–child relationship over two distinct developmental stages. Finally, in this study, not all cases analyzed were assessed entirely longitudinally, as some participants reported on trait anger and father–infant bonding concurrently. Therefore, we adjusted for wave of mediator assessment on the path between the predictor and the mediator in all analyses.

### 4.2. Conclusion

This study has implications for future research, assessment of family risk, and implementation of care. First is the promising finding that trait anger only permeates one component of the father–infant bond: patience and tolerance. Second is that trait anger in men can be used to help identify dyads where men may be at concurrent or future risk of diminished emotional connections with their infants and where fathers are at risk for heightened parenting stress during their child’s toddlerhood. This finding is critical for the facilitation and promotion of healthy father–child relationships and for protection of the child’s ongoing development and fathers’ mental health. Finally, we found evidence of both direct and mediated effects of trait anger on parenting stress in the toddler years. This suggests that if men can be engaged preconception, interventions to reduce both their angry temperament and angry reactions may promote the development of patient and tolerant emotional bonds with their infants and may lessen their parenting stress as fathers across the toddler years. However, if the transition to fatherhood is the motivator for engagement in intervention, intervention may also target the developing father–infant bond. Future research is needed to investigate whether such interventions would be feasible and effective in men who are new or soon-to-be fathers.

## Data availability statement

The data analyzed in this study is subject to the following licenses/restrictions: The datasets presented in this study are not publicly available as MAPP ethics approvals do not include participant consent for public availability of its data sets, data can be made available upon request to the corresponding author. Details about access to MAPP data files is outlined in the MAPP study cohort profile (Macdonald et al., 2021).

## Ethics statement

The studies involving human participants were reviewed and approved by Deakin University, Human Ethics Advisory Group, Faculty of Health. The patients/participants provided their written informed consent to participate in this study.

## Author contributions

LF conceptualized and designed the study with JAM, performed the statistical analysis with support from GY, CG, LG, and JAM, and wrote the draft manuscript. LF was supervised by JAM, PE, and AC. LF and JAM interpreted the results of the analyses. All authors contributed to the manuscript revision and read and approved the submitted version.

## Funding

LF was supported by a Deakin University Postgraduate Research Scholarship (DUPR). AC was supported by a Deakin University Alfred Deakin Postdoctoral Fellowship. CO was supported by a National Health and Medical Research Council Investigator Grant (APP1175086). JAM was supported by a Deakin University Mid-Career Fellowship. PE was supported by a Future Fellowship from the Australian Research Council (FT160100077).

## Conflict of interest

The authors declare that the research was conducted in the absence of any commercial or financial relationships that could be construed as a potential conflict of interest.

## Publisher’s note

All claims expressed in this article are solely those of the authors and do not necessarily represent those of their affiliated organizations, or those of the publisher, the editors and the reviewers. Any product that may be evaluated in this article, or claim that may be made by its manufacturer, is not guaranteed or endorsed by the publisher.
